# Ophthalmoparesis and Bilateral Ptosis as a Rare Manifestation of Todd's Phenomenon: Case Report and Review

**DOI:** 10.7759/cureus.26108

**Published:** 2022-06-20

**Authors:** Zaheer A Qureshi, Elina Shrestha, Pravash Budhathoki, Haider Ghazanfar, Faryal Altaf, Manjeet Dhallu

**Affiliations:** 1 Internal Medicine, Icahn School of Medicine at Mount Sinai, New York, USA; 2 Internal Medicine, BronxCare Health System, Bronx, USA; 3 Neurology, BronxCare Health System, Bronx, USA

**Keywords:** medicine, neurology, opthalmology, bilateral ptosis, atypical todd, ptosis, ophthalmoparesis, postictal deficits, todd's phenomenon, seizure

## Abstract

Todd's paresis or phenomenon (TP) is a focal weakness in a part of the body after a seizure. Seizure is an abrupt change in behavior caused by the cerebral cortex's electrical hyper-synchronization of neuronal networks. After the seizure, there is usually a transition period from the ictal state to the pre-seizure baseline level of awareness and function, referred to as the postictal period. Postictal symptoms include many systems, including sensory, motor, and psychosis. This phenomenon is named after Robert Bentley Todd, who first described it. Todd's paresis can be confused with other conditions, most commonly a stroke. Postictal ocular manifestation may be accompanied by aphasia or hemiplegia, but isolated gaze palsy is rarely reported. We are reporting a rare and first known isolated ophthalmoparesis and ptosis as postictal findings with no structural abnormalities present in imaging studies and complete resolution over three weeks on its own as an atypical postictal phenomenon. Patients with an underlying structural abnormality of the brain are more susceptible to Todd's phenomenon. Unusual manifestations of Todd's phenomenon are rare but clinically relevant and are decisive in therapeutic decision-making. Our patient presents a rare manifestation of Todd's phenomenon as ptosis and ophthalmoparesis in an elderly male with no underlying structural brain abnormalities that resolved within three weeks. Further research into the causes is needed to distinguish it from a stroke

## Introduction

Seizure disorder is common and is present in around 8-10% of the population over a lifetime [1]. A seizure is an abrupt change in behavior and motor activity caused by the cerebral cortex's electrical hyper-synchronization of neuronal networks. After the seizure, there is usually a transition period from the ictal state to the pre-seizure baseline level of awareness and function, referred to as the postictal period. The common symptoms of the postictal period are confusion and decreased alertness [2]. Postictal symptoms may include sensory, cognitive, psychiatric, and motor deficits like headaches, memory problems, decreased response, postictal depression, and psychosis [3,4]. Todd's paresis is a severe postictal motor impairment that can be confused with a stroke [5]. It can occur after a generalized or focal seizure and be unilateral or bilateral [6]. The underlying etiology of the postictal phenomenon is not correctly known. Possible mechanisms include exhaustion of neural tissues after ictal activity, elevated lactate levels, hyperperfusion during the ictal activity, or postictal cortical hypoperfusion [7-9]. Ocular manifestation like gaze palsy alone or accompanied by aphasia or hemiplegia has been reported in prior studies as a postictal phenomenon [10,11]. However, isolated gaze palsy and ocular manifestations are usually seen following stroke and are uncommon after a seizure episode. We present a 63-year-old male who developed ptosis and ophthalmoparesis following a seizure episode that persisted for three weeks and subsequently improved on its own as an atypical postictal phenomenon.

## Case presentation

Our patient is a 63-year-old man who was bought to the emergency department (ED) by the emergency medical service because of his altered mental status since morning. He had consumed alcohol all night long. His past medical history was significant for human immunodeficiency virus (HIV), cerebrovascular accident with residual unilateral lower extremity weakness, benign prostatic hypertrophy, osteoarthritis, and seizure disorder. Past surgical history was significant for appendectomy, and family history was unremarkable. As per the family members, he had been compliant with all his home medications. On general physical examination in the ED, the patient was somnolent and did not respond to verbal or noxious stimuli. He had a heart rate of 104 beats per minute, blood pressure of 130/66 mm Hg, respiratory rate of 16 breaths per minute, and temperature of 98.5 Fahrenheit. He was unable to open his eyes completely. He had normal bilateral breathing and a normal heart sound. The abdominal examination was unremarkable. Shortly after coming to the ED, he had two episodes of seizures with foaming in the mouth and fluttering of both eyes. The routine blood works were within the normal range except for mildly elevated creatine kinase and serum ethanol level of 96 mg/dl. The initial laboratory findings of the patient are presented in Table 1. 

**Table 1:** Initial laboratory results

Laboratory Parameters	Results	Reference Range
White blood cells (k/ul)	7.5	4.8-10.8
Neutrophil %	55.5	40 - 70
Hemoglobin (g/dl)	13.7	12.0-16.0
Hematocrit (%)	40.9	42.0-51.0
Platelets (150-400 k/ul)	171	150-440
Prothrombin time (PT) (seconds)	12.5	9.9 – 13.3
Partial thromboplastin time (APTT) (seconds)	29.6	27.2 – 39.6
International normalized ratio (INR)	1.08	0.85 – 1.14
Sodium (mEq/L)	140	135 - 145
Potassium (mEq/L)	3.5	3.5 – 5.0
Serum chloride (mEq/L)	104	98 - 108
Serum bicarbonate (mEq/L)	21	24 - 30
Serum glucose (mg/dl)	87	70 - 120
Blood urea nitrogen (mg/dL)	11	8 - 26
Creatinine (mg/dL)	0.8	8.5 – 10.5
Total serum calcium (mg/dL)	9.2	9 - 15
Serum magnesium (mg/dL)	1.8	1.5 – 2.7
Total bilirubin (mg/dl)	0.3	0.2 – 1.1
Direct bilirubin (mg/dl)	0.1	0.0 – 0.3
Total serum protein (g/dl)	6.8	5.8 – 8.3
Serum albumin (g/dl)	4.2	3.2 – 4.6
Alanine aminotransferase (unit/l)	18	5 – 40
Aspartate transaminase (unit/l)	26	9 – 48
Serum alkaline phosphatase (unit/l)	102	56 - 155
Serum ethanol (mg/dL)	96	< = 10 mg/dL
Serum creatine kinase (unit/L)	240	20 - 200
Absolute CD4 count (cells/ul)	844 cells/ul	490 - 1740
Human imunodeficiency virus (HIV) Ribonucleic acid (RNA) by polymerase chain reaction (PCR) copies/mL	168	Target not detected
Urine drug screen:		
Cocaine	Negative	Negative
Cannabinoid	Negative	Negative
Benzodiazepine	Negative	Negative
Methadone	Negative	Negative
Opiate	Negative	Negative
Phencyclidine (PCP)	Negative	Negative

Chest x-ray showed hazy bilateral opacities. Computed tomography (CT) scan of the head did not show any acute changes. An electrocardiogram showed atrial flutter with right bundle branch block with nonspecific ST-T wave changes. He was admitted to the floor for management of seizures and alcohol intoxication. The patient's mental status improved, and he was fully oriented to time, place, and person on day two of his hospitalization. On day three of hospitalization, he complained about the inability to open his eyes completely and worsening blurry vision. On ophthalmologic examination, he was found to have ptosis of both eyelids and limitations of extraocular movement. The patient was worked up to rule out an acute stroke and neuromuscular disorder. The results are presented in Table 2.

**Table 2:** Laboratory results for neuromuscular diseases

Laboratory Parameters	Results	Reference Range
Thyroid stimulating hormone (mIU/l)	1.78	0.40-4.50
MUSK (Muscle Specific Kinase) antibody test	Negative	Negative
Acetylcholine receptor antibody (nmol/L)	<0.30	< or =0.30 nmol/L
Lyme antibody by western blot	<0.90	< 0.90
Serum zinc (mcg/dL)	70	60 - 130
Serum copper (mcg/dl)	108	70 - 175

**Figure 1:**
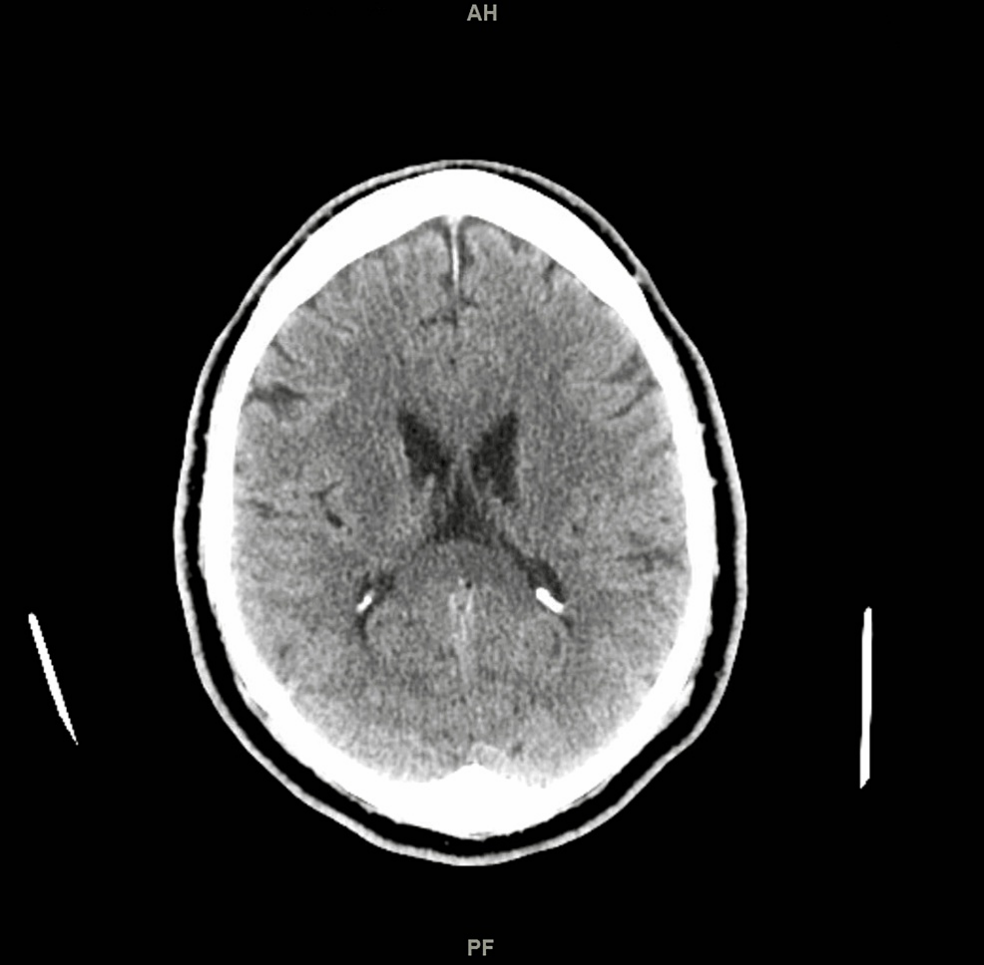
CT scan of the head showing normal architecture and no ischemic areas CT: computed tomography

**Figure 2:**
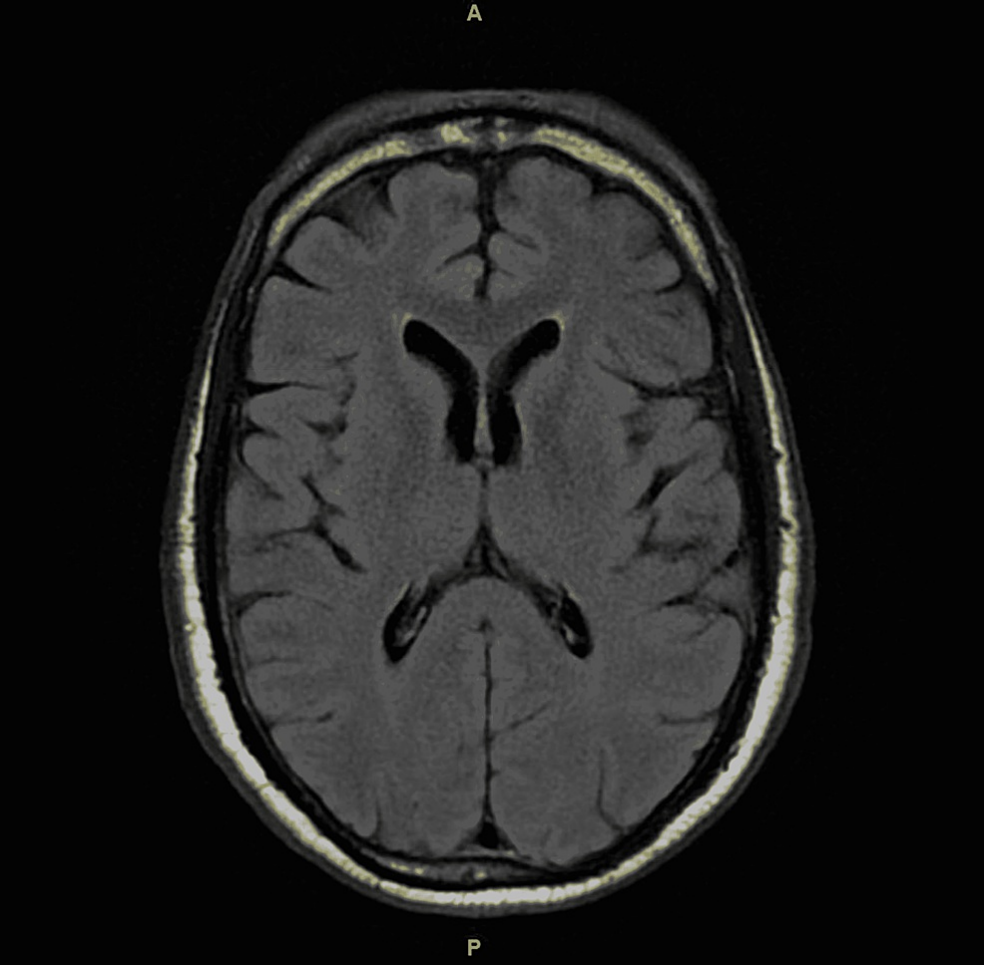
T2/FLAIR MRI of the head showing normal architecture FLAIR: fluid-attenuated inversion recovery; MRI: magnetic resonance imaging

He had a normal CT scan and magnetic resonance imaging (MRI) of the head. This has been presented in Figure 1 and Figure 2, respectively. A lumbar puncture was done, and cerebrospinal fluid (CSF) was sent for analysis. The CSF analysis has been presented in Table 3. He was counseled on alcohol use and was referred to an addiction treatment center. He was discharged with outpatient neurology follow-up. His ophthalmoparesis and ptosis resolved three weeks after being discharged. 

**Table 3:** Laboratory results of the CSP from the lumbar puncture PCR: polymerase chain reaction; CSP: cerebrospinal fluid

Cerebrospinal fluid (CSF) Analysis	Results	Reference Range
Color	Colorless	Colorless
Appearance	Clear	Clear
White blood count	3	0 - 5
Red blood cell count	1	0 - 6
Gram stain	No organism seen	No organism seen
Glucose (mg/dl)	71 mg/dl	40 - 70
Protein (mg/dL)	43	15 - 45
Lactic acid (mmoles/L)	1.4	0.6 -2.2
Cryptococcal antigen	Not detected	Not detected
Bacterial antigen	Negative	Negative
Mycobacteria culture with fluorochrome	Negative	Negative
Miscellaneous fungal culture	Negative	Negative
Viral culture	Negative	Negative
Aerobic culture	Negative	Negative
Cytomegalovirus antibody, quantitative IU/mL	<200 IU/ml	< 200
Herpes simplex virus-1 (HSV-1) and Herpes simplex virus-2 (HSV-2) quantitative, PCR	<100 copies/ml	<100 copies/ml
Venereal disease research laboratory test (VDRL) quantitative	Non-reactive	Non-reactive

## Discussion

Post-ictal state is a transient condition that occurs after seizures manifesting neurologic deficits or psychiatric symptoms, often accompanied by electroencephalography (EEG) slowing or suppression. It may last minutes to days [2]. Postictal paresis, also called Todd's paralysis is due to sustained cerebral hypoperfusion in the postictal state and occurs more frequently in patients with underlying structural lesions like ischemic stroke and hippocampal sclerosis, and atrophy [12-14]. Imaging studies have been performed at various stages ranging from ictal to postictal state to determine the degree of cortical perfusion due to conflicting reports of both cortical hypoperfusion and hyperperfusion in cases of Todd's paralysis [7,8,15]. Our patient did not have perfusion studies performed. The duration of the postictal state has been found to vary from 24 hours as per Todd to as long as 10 days [13,16].

Gaze palsy is the impairment of conjugate eye movements of both eyes in the same direction and is caused by a stroke with eye deviation to the lesion site. Isolated gaze palsy or gaze palsy with aphasia, hemiplegia, and hemianopia have been reported in prior studies [10,11]. However, visual presentations in the postictal period are scarce. Other ocular presentations include blindness, also called amaurosis, reported in 2/3 of patients with childhood occipital epilepsy of Gastaut [17,18]. The literature regarding ocular manifestation in ptosis and ophthalmoplegia is rare in the postictal period. In a prior study, postictal ptosis was reported in three patients with refractory epilepsy and left mesial temporal sclerosis ipsilaterally [19]. However, our patient's CT and MRI scans showed no structural abnormality. Our case report is the first that we, to the best of our abilities, could find in the literature to report ophthalmoparesis and ptosis as postictal findings with no structural abnormalities present on brain imaging studies and complete resolution over three weeks in the follow-up neurology visit. Detailed workup for neuromuscular junction disorder and cerebrospinal fluid analysis was done to rule out other causes of ptosis and ophthalmoparesis. However, all the tests were negative, and the patient's ptosis and ophthalmoparesis started after episodes of seizure and resolved on their own.

Video EEG studies have found slow (theta or delta) contralateral activity during seizure episodes in patients with Todd's paralysis and additional epileptiform discharges in some patients [19]. EEG performed during Todd's paralysis showed normal contralateral activity [20]. The consensus is a slow activity in the epilepsy origin area and the contralateral hemisphere in patients with Todd's paralysis. Unfortunately, no EEG was performed for our patient during ophthalmoparesis and ptosis.

## Conclusions

Todd's phenomenon in the postictal period is often confused with a stroke, and patients with an underlying structural abnormality of the brain are more susceptible to it. Todd's paresis is usually known as a cerebrovascular phenomenon. The duration of deficits varies from a few minutes to 10 days. Our study presents a rare manifestation of Todd's phenomenon as ptosis and ophthalmoparesis in an elderly male with no underlying structural brain abnormalities that resolved in three weeks. Further research into the underlying causes of Todd's paresis is needed. A physician should have a keen eye and interest in distinguishing Todd's phenomenon and acute stroke.
